# 30-Day Outcomes of Transcatheter Tricuspid Annuloplasty With the K-Clip System: A Single-Center, Observational Study

**DOI:** 10.1016/j.jacadv.2023.100671

**Published:** 2023-10-27

**Authors:** Hongfei Xu, Weidong Li, Alex Pui-Wai Lee, Shengjun Wu, Firyuza Husanova, Bifeng Wu, Yun Mou, Yanjia Gu, Miao Chen, Tingting Tao, Yiran Zhang, Junnan Zheng, Anqi Yang, Shuai Yuan, Qing Wang, Yiming Ni, Liang Ma

**Affiliations:** a Department of Cardiovascular Surgery, School of Medicine, The First Affiliated Hospital of Zhejiang University, Hangzhou, China; b Division of Cardiology, Department of Medicine and Therapeutics, Prince of Wales Hospital, Shatin, New Territories, Hong Kong; c Echocardiography and Vascular Ultrasound Center, School of Medicine, The First Affiliated Hospital of Zhejiang University, Hangzhou, China

**Keywords:** computed tomographic angiography, echocardiography, major adverse events, transcatheter tricuspid annuloplasty, transcatheter tricuspid valve repair, tricuspid regurgitation

## Abstract

**Background:**

Surgery for isolated functional tricuspid regurgitation (TR) poses a high risk. Several transcatheter approaches are being evaluated for the treatment of such patients. The K-Clip system is a percutaneous approach designed for functional TR; however, its utility remains unknown.

**Objectives:**

This study aimed to report the 30-day echocardiographic and clinical outcomes with the K-Clip system for severe TR, including changes in TR severity and NYHA functional class.

**Methods:**

Transcatheter tricuspid valve annuloplasty was performed in 39 patients with intermediate or high surgical risk who underwent the K-Clip system. The right internal jugular vein procedure was performed with annuloplasty guided by fluoroscopy and echocardiography. The primary outcomes were clinical success and all-cause mortality at the 30-day follow-up.

**Results:**

The K-Clip was successfully implanted in all cases, with 1 to 3 devices deployed. At the 30-day follow-up, none of the patients had died. TR severity was reduced by at least one grade in all patients. There were no severe procedural or 30-day adverse events, except for 1 new pacemaker implantation. The proportion of NYHA class III-IV patients decreased from 79.5% to 5.1%, and the ascites disappeared. The 6-minute walk distance increased by 78 m (*P* < 0.05), and the Kansas City Cardiomyopathy Questionnaire score improved by 11 points (*P* < 0.05).

**Conclusions:**

The K-Clip device is practical, safe, and effective for patients with severe TR. A 30-day reduction in TR and enhanced cardiac function and quality of life were associated with transcatheter tricuspid annuloplasty using the K-Clip device, according to short-term follow-up studies. (Confirmatory Clinical Study of Treating Tricuspid Regurgitation With K-Clip TM Transcatheter Annuloplasty System [TriStar]; NCT05173233)

Moderate-to-severe tricuspid regurgitation (TR), the most prevalent disease on the right side of the heart, has become a growing public health crisis. TR occurs in approximately 4% of elderly patients and is more common in women^1^; most of them are unable to receive appropriate care. Although primary (organic) TR is uncommon, secondary (functional) TR (FTR) is dominant, accounting for 80% to 90% of TR.^2^ The tricuspid valve (TV) is unaffected by organic disorders; however, FTR may occur in the context of left heart valve disease, pulmonary hypertension, atrial fibrillation, or other diseases.^3^ Although mitral valve surgery can somewhat improve FTR to a certain extent, untreated severe TR causes long-term heart failure and other consequences, affecting the quality of life and increasing mortality.^4^

Although medication therapy alone is inadequate, the clinical therapies for TR include conservative drug treatments and surgery. Surgical correction of the FTR, particularly tricuspid valvuloplasty similar to left heart surgery, can significantly enhance the long- and short-term prognoses of patients.^5^ Nevertheless, investigations have discovered that the high recurrence rate and mortality after tricuspid valvuloplasty are severe and intractable issue.^6^ Solitary TV surgery is a risky contemporary valve procedure, with a mortality rate of 8.8% to 9.7%.^5^

Therefore, there is a need for less-invasive treatments. Valve repair and replacement are the major components of TV transcatheter therapy and are currently in the early stages of clinical verification. Less trauma, minimal procedural risk, few complications, quick recovery, and high patient acceptability are benefits of interventional therapy for TR. Patients with severe TR who cannot undergo surgery are good candidates.

The transcatheter TV repair techniques can be divided into edge-to-edge leaflet repair, annuloplasty, and regurgitation orifice filling.^7^ The main pathology in the majority of patients with TR is annular dilatation, and minimally invasive catheter-based devices are designed to shrink the size of tricuspid annular dimensions. In the context of the surging development of transcatheter TV intervention strategies, the results of the epoch-making randomized controlled trial TRILUMINATE Pivotal brought confidence to TV interventional therapies.^8^ The existing devices still have a number of common issues, including difficult operation procedures, steep learning curves, and a lack of large-scale clinical testing to confirm their efficacy. Transcatheter TV repair devices should be simple to use and widely available. Therefore, the K-Clip system was developed. The K-Clip transcatheter tricuspid annuloplasty system (Huihe Medical Technology) is a percutaneous approach designed for FTR through anchors on the annulus tissue utilizing a corkscrew, which lowers the dimensions of the tricuspid annulus by folding and clamping the tricuspid annulus tissue with a rigid clamping device. The K-Clip has potential benefits for TR caused by tricuspid annulus dilatation, especially in patients with large valve leaflet gaps (coaptation gaps >10 mm). Pan et al^9^ introduced the working principle and efficacy of the K-Clip in animals. The device concept and details of the implantation process have been detailed previously. Herein, we reported 39 patients completed in our center from July to October 2022. All patients were at intermediate or high surgical risk (Tri-Score ≥4)^10^ and concluded a short-term 30-day follow-up.

## Methods

### Device description

A catheter-based treatment for TR, the K-Clip tricuspid annuloplasty system, consists of an anchor device, clip device, delivery system, and holder system.^9^ The K-Clip precisely reaches the desired location of the TV area through the internal jugular vein approach. The anchor and clip devices are similar in shape to a crocodile mouth, which bites the enlarged TV annulus tissue and shrinks the TV annulus. The circumference of the TV is reduced, thus bringing together the TV leaflets in areas of malcoaptation and enhancing sufficient valve closure. Four clip sizes are available with arm lengths of 12, 14, 16, and 18 mm. All patients provided signed informed consent. This study was registered at ClinicalTrials.gov (NCT05173233).

### Patient selection

Patients aged >60 years had an FTR severity grade ≥4 and NYHA functional class ≥II. The heart team identified patients at intermediate or high surgical risk with a Tri-Score ≥4 and left ventricular ejection fraction (LVEF) ≥40%. The etiology of FTR included TR due to right atrial disease and/or right ventricular (RV) dysfunction. The causes of the latter included RV cardiomyopathy, RV myocardial infarction, left valvular disease, and congenital heart disease. The exclusion criteria are listed in Supplemental Table 1. The local ethics committee and Chinese Ministry of Health approved the study (QL2100003).

### Preoperative screening assessment

In addition to evaluating the patients’ basic information prior to surgery, symptoms, past medical history, and heart-related surgery history were obtained to analyze the 6-minute walk distance (6MWD), Kansas City Cardiomyopathy Questionnaire (KCCQ), blood indicators such as blood routine, biochemistry, B-type natriuretic peptide, N-terminal pro-B-type natriuretic peptide, and cardiac function. Transthoracic echocardiography (TTE), transesophageal echocardiography (TEE), and coronary computed tomographic angiography (CTA) are among the imaging techniques.

### Preprocedural imaging TTE

**Figure 1:**
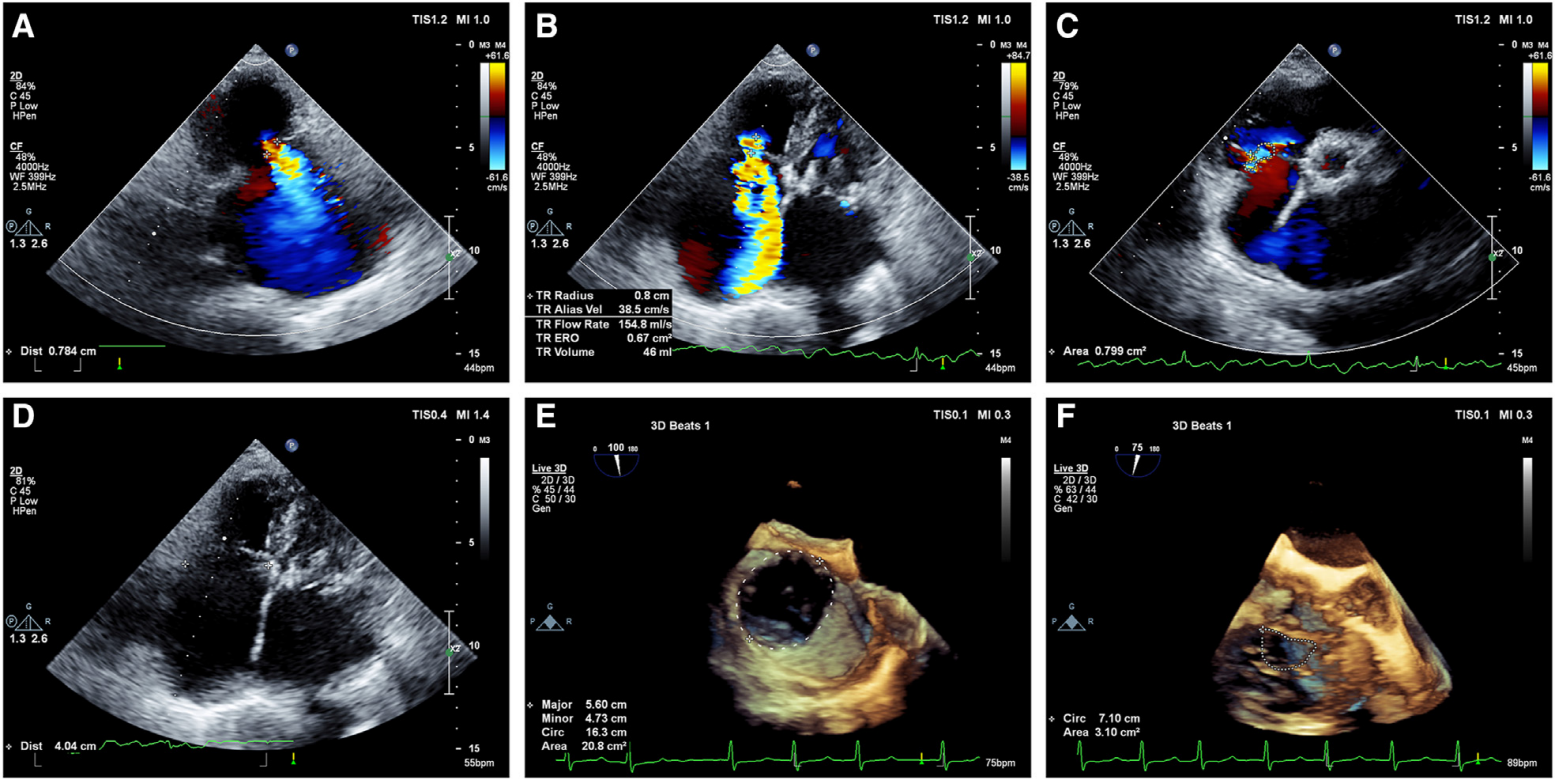
**Preprocedural TTE and TEE** (A) TR vena contracta width is measured from TTE (width, 7.8 mm). (B) PISA estimated Doppler volume method EROA and regurgitant volume are measured from TTE (EROA, 67 mm^2^; regurgitant volume, 46 mL). (C) The vena contracta area is measured from TTE (area, 79.9 mm^2^). (D) The septolateral diameter of the tricuspid valve annulus is measured from TTE (diameter, 4.0 cm). (E) The maximum circumference and area of the tricuspid annulus are measured from 3D TEE in diastole (circumference, 16.3 cm; area, 20.8 cm^2^). (F) The gap shape is shown from 3D TEE in systole (gap area, 3.1 cm^2^). 3D = 3-dimensional; EROA = effective regurgitant orifice area; PISA = proximal isovelocity surface area; TEE = transesophageal echocardiography; TR = tricuspid regurgitation; TTE = transthoracic echocardiography.

Echocardiography remains the most important imaging modality for evaluating the etiology and severity of TR. We conducted a TTE evaluation for each patient, targeting TV-related data collection following the American Society of Echocardiography guidelines.^11^^,^^12^ Various parameters, such as the degree of TR, right atrial superior to inferior and left to right diameters, pulmonary systolic blood pressure, TR vena contracta width (VCW) (Figure 1A), proximal isovelocity surface area estimated Doppler volume technique effective regurgitant orifice area (EROA) (Figure 1B), vena contracta area (Figure 1C), regurgitant volume (Figure 1B), septolateral diameter of the TV annulus (Figure 1D), anteroposterior diameter of the TV annulus, and the diameter of the RV (basal segment), were computed from echocardiography (EPIQ 7C, Philips Healthcare). The fractional area alterations of the RV (fractional area change [FAC]), inferior vena cava (IVC) width, IVC variability, hepatic vein systolic flow reversal, tricuspid annular plane systolic excursion (TAPSE), degree of mitral valve regurgitation, and ejection fraction were analyzed in the echocardiography core laboratory. All echocardiograms were evaluated at an independent core laboratory in Shanghai, China. We reported the grade of TR using a 6-grade scheme of 1 (trace or mild), 2 (moderate), 3 (significant/moderate-severe), 4 (severe), 5 (massive), or 6 (torrential), based on the recently updated echocardiographic assessment of interventional therapy for TR by Hahn et al.^13^

### Preprocedural imaging TEE

TEE and focused TV imaging employing 3-dimensional (3D) modalities were performed for each patient prior to surgery. During diastole, 3D imaging of the TV was performed to determine the maximum circumference (Figure 1E) and area of the tricuspid annulus (Figure 1E). During systole, the gap shape (Figure 1F), regurgitation location, and regurgitation volume of the TV orifice were observed.

### Preprocedural imaging CTA

**Figure 2:**
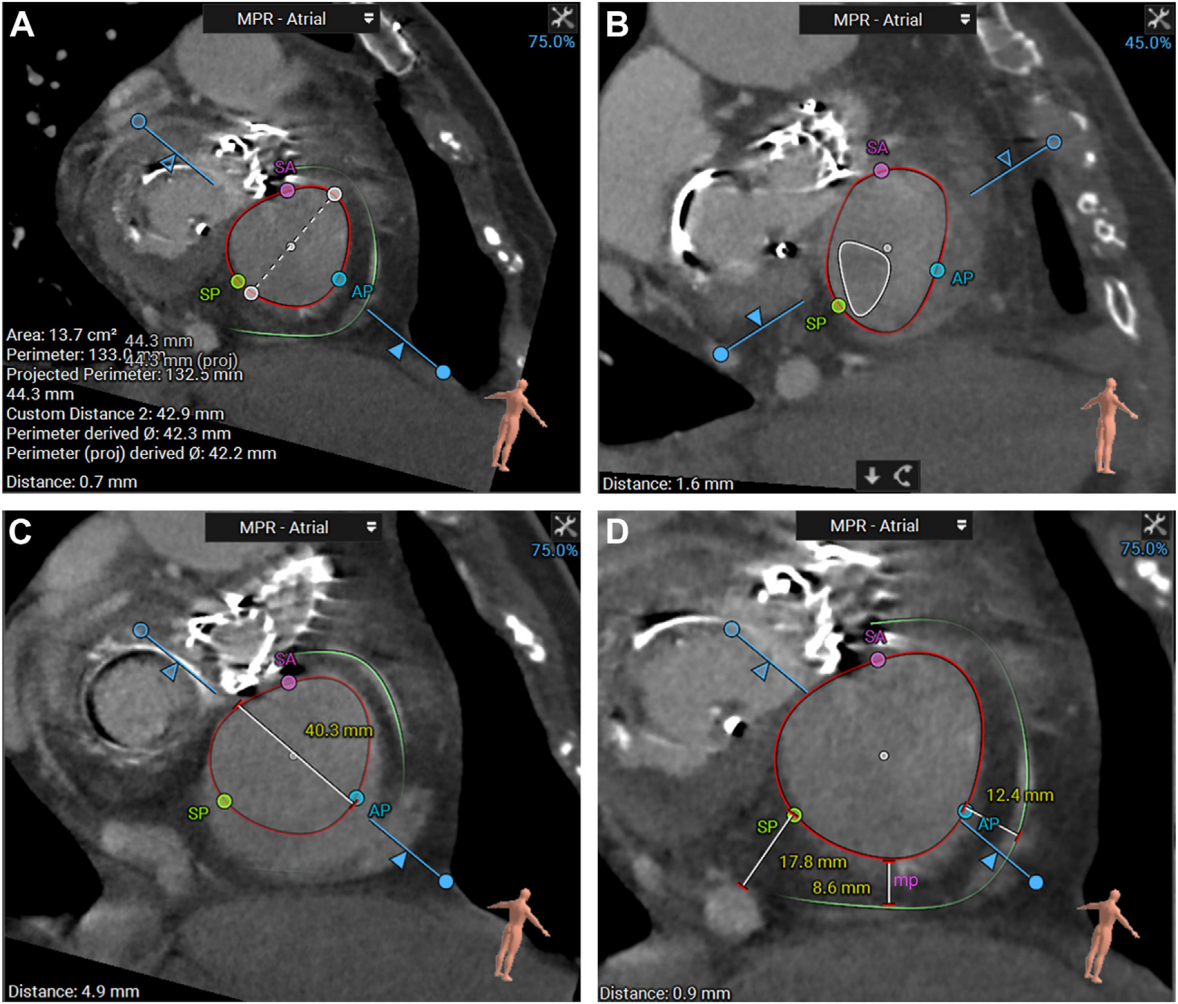
**Preprocedural CTA** (A) Tricuspid valve orifice area and tricuspid annulus circumference in diastole. (B) TR orifice shape in systole. (C) Septolateral diameter of the tricuspid valve annulus in diastole. (D) Annulus to right coronary artery distance in the location of the posteroseptal commissure (sp), the midpoint of the posterior valve annulus (mp), and anteroposterior commissure (ap), respectively. CTA = computed tomographic angiography; TR = tricuspid regurgitation.

All patients underwent electrocardiography-gated CTA (Philips Healthcare) following a dedicated TV protocol using a 256-slice system. Using a 2-stage technique, intravenous nonionic contrast (iodixanol) was administered: 100% contrast at 4.5 mL/s for 10 second, followed by 35%/65% contrast/saline mixture at 4.5 mL/s for 20 second (total volume of contrast medium, 76 mL). Data were stored for every 10% of cardiac cycles. Computed tomographic assessment of the tricuspid annulus and right coronary artery (RCA) information was sustained by a semiautomated software-based approach.^14^ The recorded images were employed to compute the TV orifice area (Figure 2A), tricuspid annulus circumference (Figure 2A), TR orifice shape (Figure 2B), septolateral diameter of the TV annulus (Figure 2C), landing zone for the K-Clip, annulus to RCA distance such as posteroseptal commissure (Figure 2D), midpoint of the posterior valve annulus (Figure 2D), and anteroposterior commissure (Figure 2D), and evaluate whether a right-dominant circulation was present (3 mensio valves, Pie Medical Imaging).

### Procedure

**Figure 3:**
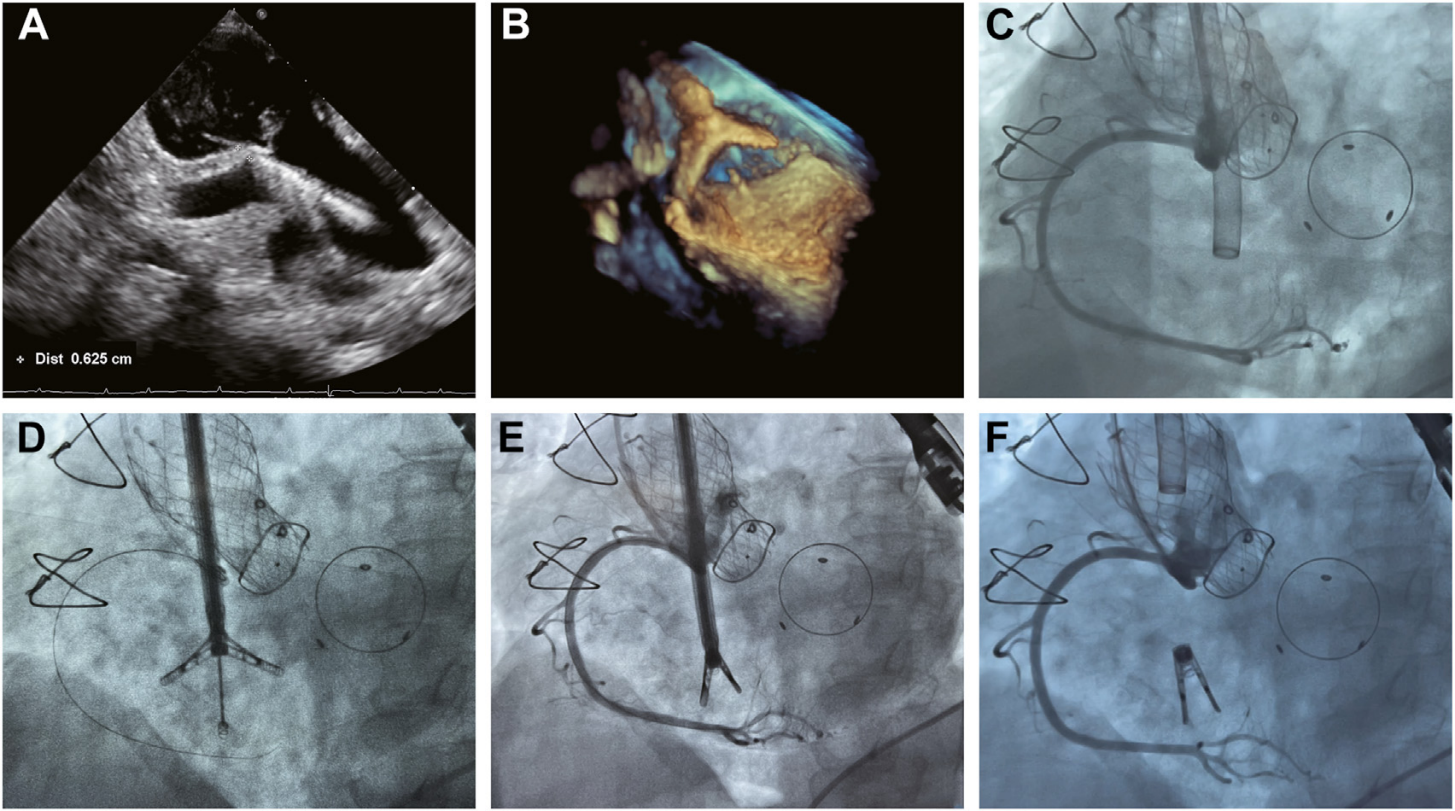
**Case Example of K-Clip System Deployment** (A) The corkscrew is drilled into the annulus under TTE guidance. (B) The clip arm is rotated to make it flush with the annulus under the 3D guidance of TEE. (C) The deflectable outer sheath is placed, and right coronary angiography is performed. (D) The corkscrew with the annulus tissue is raised outward to the unfolded clip arms. (E) Right coronary angiography is used to evaluate the presence of coronary stenosis and clip stability. (F) After release, angiography is performed again to assess the vascular stenosis. 3D = 3-dimensional; TEE = transesophageal echocardiography; TTE = transthoracic echocardiography.

Following single-lumen endotracheal tube intubation, patients were kept in the supine position while undergoing RCA angiography and placement of a coronary guidewire implanted through the right femoral artery to help define the TA plane fluoroscopically. In all cases, the TV was approached using the right internal jugular vein technique after complete heparinization with a goal-activated clotting time of 250 to 300 second. The deflectable outer sheath (16-F) was placed with the tip in the middle RA (Figure 3C). The deflectable inner sheath (15-F), including the anchor and clip, was placed into the outer sheath and emerged to the superior vena cava. The fissure distribution of the TR was assessed using 3-day TEE guidance, and the angle of the delivery system was modified so that the corkscrew was perpendicular to the target annulus. The corkscrew was drilled into the annulus with a beating heart using TTE guidance, and the firmness was verified using a pull test to grip the annulus (Figure 3A). Under the 3D guidance of TEE and digital subtraction angiography, the clip arms were unfolded, and the arm was rotated to make it flush with the annulus (Figure 3B) and then advanced in the direction of the annulus until the clip arms were close to the annulus tissue. Subsequently, the corkscrew with the annulus tissue was raised outward (Figure 3D), the clip arms were closed, and then the annulus and coaptation gaps were reduced. Right coronary angiography was performed to simultaneously evaluate the presence of coronary stenosis and clip stability (Figure 3E). If the assessment was satisfactory (TR reduction grade of ≥2), the clip was removed from the delivery sheath, and TEE assessment and angiography were performed again to assess vascular stenosis (Figure 3F). Otherwise, a new clip was implanted until satisfactory TEE results were obtained. Before release, the delivery system and clips can be retrieved and removed. All patients received oral anticoagulants (aspirin plus clopidogrel or warfarin alone) for 6 months after surgery.

### Clinical outcomes

All-cause mortality, stroke, transient ischemic attacks, myocardial infarction, significant/life-threatening bleeding (defined as Bleeding Academic Research Consortium type ≥3a), acute kidney injury (defined as the need for unplanned dialysis or renal replacement therapy), renal insufficiency (estimated glomerular filtration rate <60 mL/min/1.73 m^2^), new pacemaker implantation, ventricular arrhythmia, pulmonary embolism, conversion to open heart surgery, procedure-associated RCA stenosis, endocarditis, and device dislocation are among the safety events that are monitored by an independent clinical event committee. Patients were assessed at baseline, in-hospital, postprocedure, and 30 days after the procedure. Clinical and functional endpoints were evaluated based on the clinical characteristics of heart failure (ascites and peripheral edema), all-cause mortality, NYHA, KCCQ, and 6MWD.

### Statistical analysis

The mean ± SD or median (IQR) were the terms used to characterize continuous variables. Categorical variables were denoted as frequencies and percentages. Paired data served as the basis for all comparisons. Paired Student’s *t-*test was used to determine the differences between the 2 groups. Statistical significance was defined as *P* < 0.05. Statistical analyses were conducted using SPSS 20.0 (IBM) and GraphPad Prism 7.0.

## Results

### Baseline patient characteristics

Between July and October 2022, we screened 52 individuals with severe TR due to age, pulmonary hypertension, or other causes. Finally, 39 patients from a single study center in mainland China who had symptomatic severe TR (TR severity grade of ≥4) underwent surgery. Table 1 shows the baseline clinical characteristics of patients. The mean age of the patients was 73.0 (IQR: 66-76) years, 69.2% of the patients were female, 48.7% patients had a history of previous left heart valve replacement surgery, most patients had atrial fibrillation/flutter (n = 36, 92.3%), some patients experienced a stroke before surgery (n = 3, 7.7%), and 79.5% of the patients were NYHA functional class III/IV. Other comorbidities comprised of hypertension (n = 12, 30.8%), diabetes (n = 5, 12.8%), renal insufficiency (n = 10, 25.6%), and a median Tri-Score of 4 (n = 39). In addition, 71.8% of the patients had ascites or peripheral edema prior to the surgery; the baseline KCCQ was 64.9 ± 15.1, and the 6MWD was 284.6 ± 111.6 m. Nineteen patients had a history of cardiac surgery, such as mitral valve surgery (n = 17, 43.6%), aortic valve surgery (n = 13, 33.3%), coronary artery bypass grafting (n = 2, 5.1%), pacemaker leadless (n = 1, 2.6%), and radiofrequency ablation (n = 2, 5.1%). Supplemental Table 2 summarizes the baseline blood test data.

**Table 1:** Baseline Characteristics of the Patients or Cohort (N = 39)

Baseline clinical characteristics	
Age, y	73.0 (66-76)
Female	27 (69.2%)
Body surface area, m^2^	1.7 (1.6-1.7)
Hypertension	12 (30.8%)
Diabetes mellitus	5 (12.8%)
Atrial fibrillation	36 (92.3%)
Previous stroke	3 (7.7%)
Chronic kidney disease, eGFR <60 mL/min (mL/min/1.73 m^2^)	10 (25.6%)
Previous admission for heart failure	28 (71.8%)
Kansas City Cardiomyopathy Questionnaire	64.9 ± 15.1
6-min walk distance, m	284.6 ± 111.6
Tri-Score	4.0 (4.0-5.0)
NYHA functional class	
I	0 (0%)
II	8 (20.5%)
III	29 (74.4%)
IV	2 (5.1%)
Clinical features of heart failure	
Ascites	3 (7.7%)
Peripheral edema	28 (71.8%)
Previous cardiac surgery	
Previous ViV-TAVI	2 (5.1%)
Previous mitral valve surgery	17 (43.6%)
Previous aortic valve surgery	13 (33.3%)
Previous tricuspid valve surgery	0 (0%)
Previous CABG	2 (5.1%)
Previous PCI	0 (0%)
Previous pacemaker lead	0 (0%)
Previous pacemaker leadless	1 (2.6%)
Previous radiofrequency ablation	2 (5.1%)

Values are median (IQR), n (%), or mean ± SD.

CABG = coronary artery bypass grafting; eGFR = estimated glomerular filtration rate; PCI = percutaneous coronary interventions; ViV-TAVI = valve-in-valve transcatheter aortic valve implantation.

The echocardiographic data at baseline are listed in Supplemental Table 3. All data were obtained from TTE or TEE. All patients exhibited a baseline TR severity grade of ≥4 (grade 4, 56.4%; grade 5, 20.5%; grade 6, 23.1%) and mitral regurgitation grade of ≤3. The mean TR VCW was 12.7 ± 5.7 mm, proximal isovelocity surface area EROA was 99.5 ± 77.8 mm^2^, regurgitant volume was 71.1 ± 38.7 mL, diastolic TV opening area was 15.0 ± 5.2 cm^2^, TV ring circumference was 137.0 ± 23.1 mm, FAC was 43.4% ± 7.9%, IVC diameter was 22.0 ± 7.1 mm, TAPSE was 18.4 ± 3.7 mm, and mean LVEF was 62.2% ± 7.8%.

Supplemental Table 4 displays the baseline CTA results determined by 3mensio valves software. The mean diastolic TV opening area was 1714.8 ± 481.9 mm^2^, the TV ring circumference was 149.4 ± 20.7 mm, and the left to right diameter of the septal valve ring was 46.8 ± 9.3 cm. The potential number of the landing zone (plication area) for the K-Clip was 1.9 ± 0.7, and the distances of the annulus–RCA in 3 anatomical sites (potential landing zones), namely, posteroseptal commissure, midpoint of the posterior valve annulus, and anteroposterior commissure, were 15.5 ± 5.2 mm, 6.4 ± 3.5 mm, and 8.5 ± 3.2 mm, respectively.

### Procedural findings

Table 2 lists the key features of the procedure. The K-Clip was successfully implanted in all 39 patients with 1 to 3 devices installed, with a mean of 1.5 implants per patient (one implant in 56.4%, 2 implants in 38.5%, and 3 implants in 5.1% of patients). The treatment took a mean of 137.6 ± 55.6 minutes. The mean fluoroscopy time was 43.1 ± 15.3 minutes, the volume of contrast medium was 69.7 ± 28.2 mL, and there were no complications such as conversion to open heart surgery, procedure-associated RCA stenosis (≥50%), device dislocation into right atrium, or death during surgery. The most frequently used clip size was size 16 (n = 31, 53.4%), the most common landing zone for K-Clip placement was the posteroseptal commissure (56.4%), and TR was reduced by at least 1 grade in all patients (2.6% reduced by 1 grade, 51.3% reduced by 2 grades, 38.4% reduced by 3 grades, and 7.7% reduced by 4 grades). The length of hospital stay was 8.00 (IQR: 7.0-10.5) days.

**Table 2:** Procedural Findings (N = 39)

Procedural findings	
Procedure time, skin-to-skin, min	137.6 ± 55.6
Fluoroscopy time, min	43.1 ± 15.3
Contrast media, mL	69.7 ± 28.2
Successful implantation	39 (100%)
Conversion to open heart surgery	0 (0%)
Massive pericardial effusion	0 (0%)
Procedure-associated right coronary artery stenosis, ≥50%	0 (0%)
Device dislocation into the right atrium	0 (0%)
Mortality	0 (0%)
K-Clip size	
12	3 (5.2%)
14	19 (32.8%)
16	31 (53.4%)
18	5 (8.6%)
Number of K-Clip	
0	0 (0%)
1	22 (56.4%)
2	15 (38.5%)
3	2 (5.1%)
K-Clip location	
Anteroseptal commissure	0 (0%)
Anteroposterior commissure	0 (0%)
Posteroseptal commissure	22 (56.4%)
Anteroposterior + posteroseptal	13 (33.4%)
The midpoint of the posterior + posteroseptal	2 (5.1%)
Other combinations	2 (5.1%)
TR reduction at the procedure	
1 grade	1 (2.6%)
2 grades	20 (51.3%)
3 grades	15 (38.4%)
4 grades	3 (7.7%)
5 grades	0 (0%)
In-hospital outcomes	
Success	39 (100%)
ICU stay, d	2 (1-2.5)
Length of stay, d	8 (7-10.5)

Values are median (IQR), n (%), or mean ± SD. The severity of TR was assigned a grade of 1 (trace or mild), 2 (moderate), 3 (significant/moderate–severe), 4 (severe), 5 (massive), or 6 (torrential).

ICU = intensive care unit; TR = tricuspid regurgitation.

### 30-Day outcomes of the population

**Figure 4:**
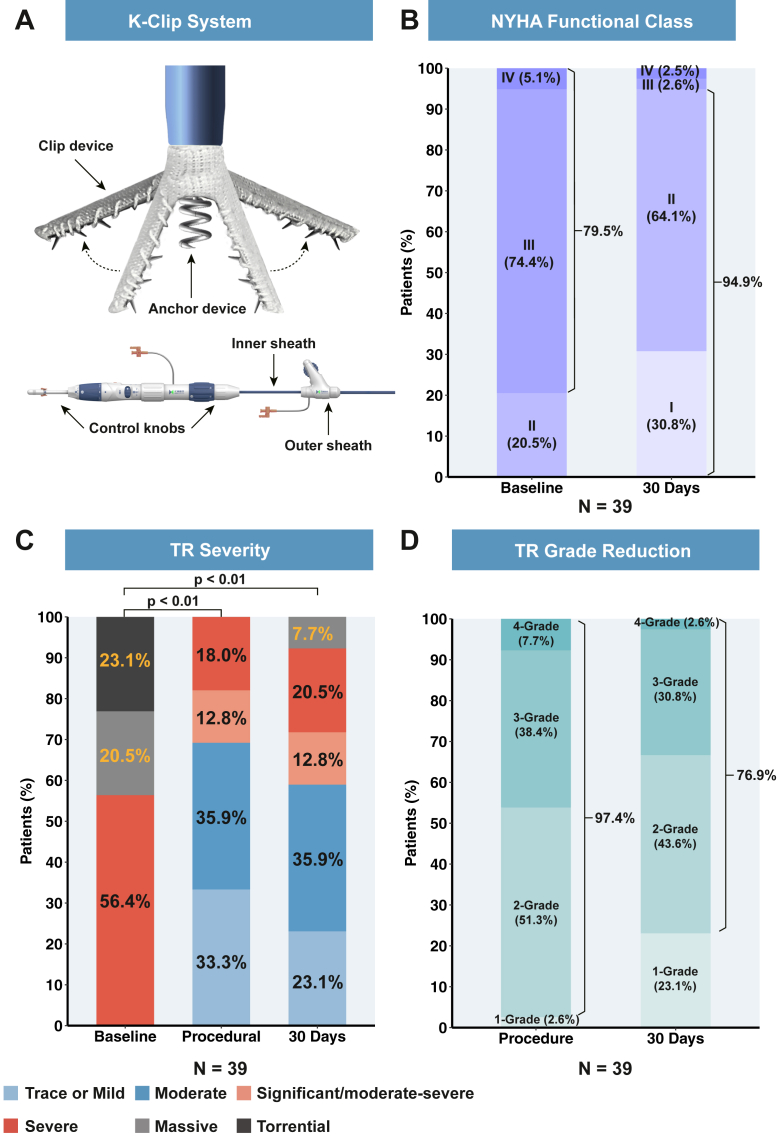
**The K-Clip Transcatheter Tricuspid Annuloplasty System** (A) The K-Clip tricuspid annuloplasty system. The system comprises an anchor device, clip device, outer (16-F) and inner (15-F) sheaths, and control knobs. Control knobs are adjusted so that the distal end of the inner sheath and anchor device is parallel to the annulus and pointing to the target area. The successful capture of the annulus tissue reduces the annulus area and circumference. (B) NYHA functional class changes from baseline to 30 days after surgery. (C) TR severity at baseline, during the procedure, and 30 days after the procedure in paired analysis. (D) Grade reduction of TR severity at the procedure and 30 days after surgery. The *P* values for categorical variables are derived from the Wilcoxon signed-rank test. Values are mean. TR = tricuspid regurgitation.

**Figure 5:**
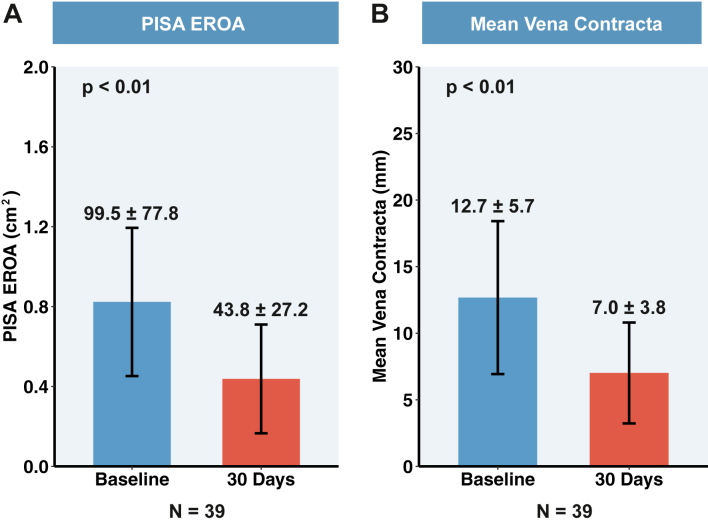
**Reduction in TR From Baseline to 30 Days With the K-Clip System** (A) The mean PISA EROA decreases from 99.5 ± 77.8 mm^2^ to 43.8 ± 27.2 mm^2^. (B) The mean TR vena contracta width decreases from 12.7 ± 5.7 mm to 7.0 ± 3.8 mm. The *P* values for continuous variables are derived from paired Student’s t-tests. Values are mean ± SD. The error bars represent the 95% CIs. EROA = effective regurgitant orifice area; PISA = proximal isovelocity surface area; TR = tricuspid regurgitation.

Figures 4 and 5 show that at the 30-day postoperative follow-up, 23.1%, 43.6%, and 33.3% of patients achieved reductions in TR grades of 1, 2, and at least 3, respectively. TTE measurements demonstrated a tricuspid regurgitant volume from 71.1 ± 38.7 mL to 36.2 ± 23.4 mL (*P* < 0.05), with a significant mean reduction of 49.0%; TV EROA from 99.5 ± 77.8 cm^2^ to 43.8 ± 27.2 cm^2^ (*P* < 0.05), with a significant median reduction of 55.6%; TR VCW from 12.7 ± 5.7 mm to 7.0 ± 3.8 mm (*P* < 0.05), with a mean reduction of 44.6%; IVC diameter from 22.0 ± 7.1 mm to 17.8 ± 7.6 mm (*P* < 0.05), with a significant mean reduction of 19.3%; and RV diameter base from 45.8 ± 7.6 mm to 40.5 ± 8.4 mm (*P* < 0.05), with a significant mean reduction of 11.8%. The FAC, TAPSE, and LVEF exhibited no significant difference.

**Figure 6:**
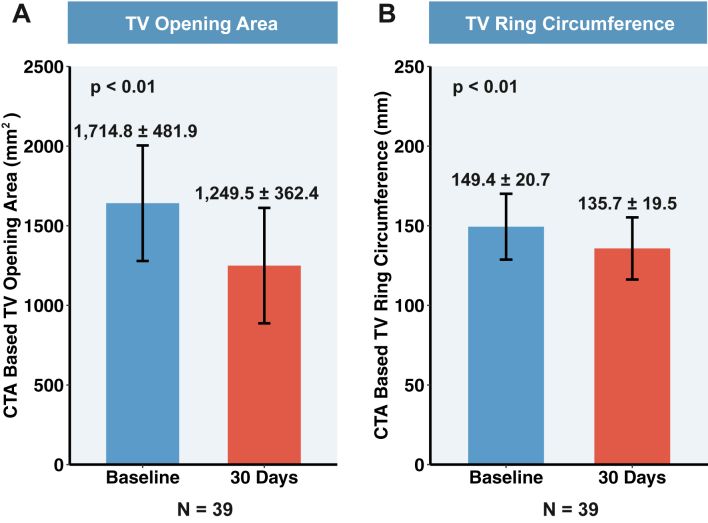
**The K-Clip System Reduces the TV Area and Ring Circumference From Baseline to 30 Days** (A) The diastolic TV opening area decreases from 1,714.8 ± 481.9 mm^2^ to 1,249.5 ± 362.4 mm^2^ at 30 days. (B) The TV ring circumference decreases from 149.4 mm ± 20.7 to 135.7 ± 19.5 mm at 30 days. The p values for continuous variables are derived from paired Student’s *t*-tests. Values are mean ± SD. The error bars represent the 95% confidence intervals. CTA = computed tomographic angiography; TV = tricuspid valve.

The diastolic TV opening area computed by CTA decreased at 30 days from 1,714.8 ± 481.9 mm^2^ to 1,249.5 ± 362.4 mm^2^ (*P* < 0.01), the TV ring circumference decreased from 149.4 ± 20.7 mm to 135.7 ± 19.5 mm (*P* < 0.01) (Figure 6), and the septolateral diameter of the TV ring decreased from 46.8 ± 9.3 mm to 32.0 ± 6.5 mm (*P* < 0.05).

**Figure 7:**
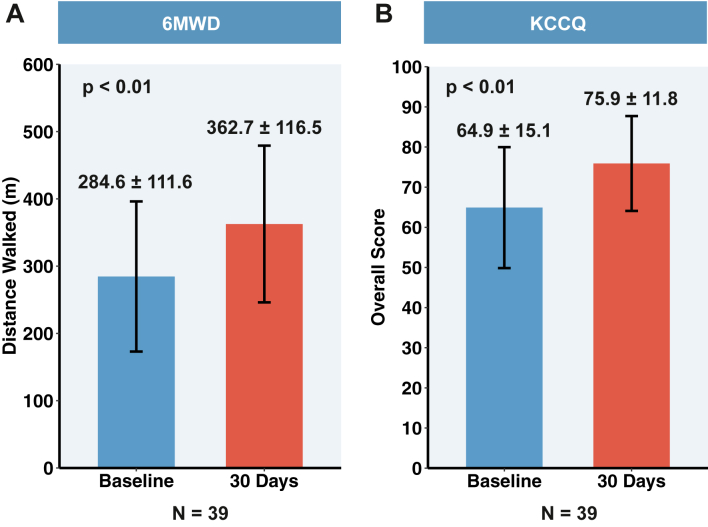
**The K-Clip System Improves the 6MWD and KCCQ Scores From Baseline to 30 Days** (A) The mean 6MWD improves from 284.6 ± 111.6 m to 362.7 ± 116.5 m at 30 days. (B) The mean KCCQ score improves from 64.9 ± 15.1 to 75.9 ± 11.8. The *P* values for continuous variables are derived from paired Student’s *t*-tests. Values are mean ± SD. The error bars represent the 95% CIs. 6MWD = 6-minute walk distance; KCCQ = Kansas City Cardiomyopathy Questionnaire.

Figures 4 and 7 show that significant clinical improvements were observed in the NYHA functional class after a 30-day follow-up (94.9% of patients had NYHA class I/II, and the proportion of class III–IV patients decreased from 79.5% to 5.1%). The 6MWD improved from 284.6 ± 111.6 m to 362.7 ± 116.5 m (*P* < 0.01), and the KCCQ score improved from 64.9 ± 15.1 to 75.9 ± 11.8 (*P* < 0.01).

All 39 patients attained clinical success with the K-Clip repair system (both implantation and success rates were 100% [Table 2], and implantation success was defined as a reduction in TR of at least 1 grade). The 30-day primary safety endpoint of the major adverse event (MAE) rate was 2.6%. Except for the implantation of a new pacemaker, no deaths, severe right coronary compression, need for intervention, myocardial infarction, stroke, significant/life-threatening bleeding, acute kidney injury, rehospitalization for heart failure, or other serious complications occurred during the 30-day follow-up (syncope one time at home 2 weeks after surgery, suggesting bradycardia). In addition to having a pacemaker implanted, 2 patients had prolonged hospitalization (>2 weeks): one had a lung infection, and the other had gastrointestinal bleeding. Both patients recovered and were discharged after conservative treatment.

## Discussion

To the best of our knowledge, this is the first study to report the 30-day results of a single-center, observational, first-in-human experience study testing transcatheter tricuspid annuloplasty with the K-Clip system for treating severe functional TR. The short-term follow-up displayed promising outcomes, including the following (Central Illustration): 1) the K-Clip was a safe and reliable surgical procedure, 100% of the clips were successfully implanted, and the decrease in the grade of TR and reduction in the area of the valve ring were substantial and durable; 2) the quality of life and cardiac function improved; 3) the incidence of adverse events was low, with an MAE rate of 2.6%, which is lower than that of the CLASP TR EFS^15^ results and close to the TRILUMINATE Pivotal^8^ results; and 4) the RV remodeling was noted—30 days after surgery, the diameter of the RV substantially decreased.

**Central Illustration:**
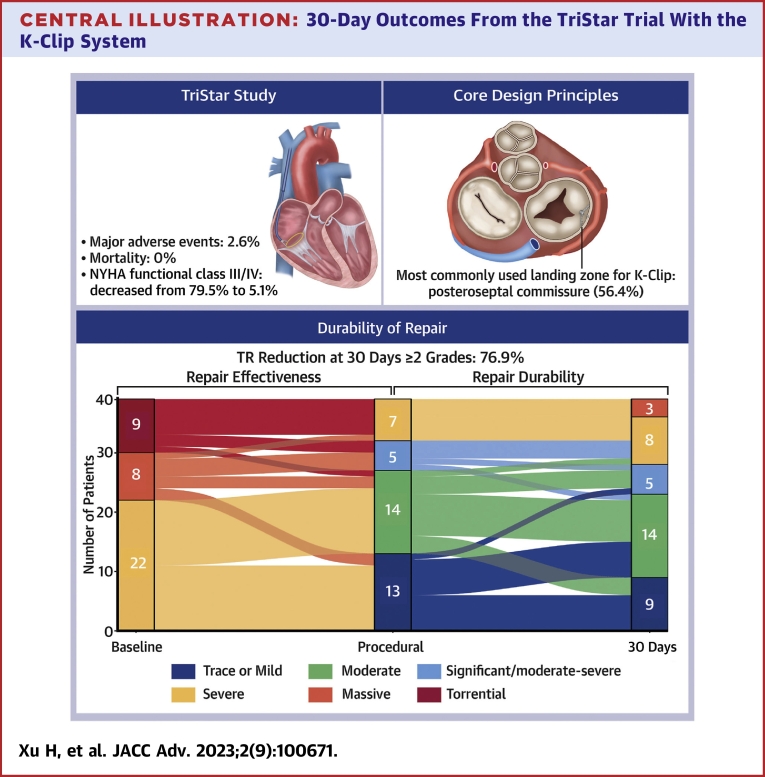
**30-Day Outcomes From the TriStar Trial With the K-Clip System** Transcatheter tricuspid valve repair using the K-Clip system is technically feasible, safe, and effective. The major adverse event and mortality rates are low. The most commonly used landing zone for the K-Clip is posteroseptal commissure (56.4%), with high coronary safety. Short-term follow-up shows that the K-Clip system is associated with a 30-day reduction in TR and improved cardiac function and quality of life. TR reduction is observed to be at least grade 1 in all patients. This effect persists at follow-up 30 days postoperatively, with a reduction of ≥2 grades in 76.9% of patients. TR = tricuspid regurgitation.

All patients experienced a decrease in the TR of at least one grade when the K-Clip system was successfully implanted. At follow-up 30 days after surgery, this effect remained, with a decrease of ≥2 grades in 76.9% of patients. After annular reduction from 3D imaging, even though complete coaptation of the TV leaflets may not be accomplished or the degree of TR remained at grade 4, the relative reduction in EROA and TR was significant to lead to considerable clinical and hemodynamic improvement, linked to the enhancement in the stroke volume of the RV and increase in forward blood flow, which then caused increases in the preload of the left heart system and contractility. According to the CTA measurement data, the distance of the RCA from the tricuspid annulus changed in a wave-like pattern from its proximal end to its distal end. Thus, from the viewpoint of coronary safety, the potential landing zones from high to low safety variables are the posteroseptal commissure, anteroposterior commissure, and midpoint of the posterior valve annulus.

A strength of this study is that no deaths, massive pericardial effusion, or cases of conversion to thoracotomy were recorded at 30 days of follow-up. The current results are somewhat equivalent to those of earlier clinical studies on transcatheter TV interventional therapy,^16^, ^17^, ^18^, ^19^, ^20^ despite the short follow-up time, which gives us more confidence in our long-term efficacy outcomes. A 30-day short-term follow-up revealed significant clinical improvement in addition to minimal mortality and serious bleeding problems. The KCCQ score, 6MWD, and proportion of patients with NYHA functional class I/II significantly increased within 30 days, as did the symptoms of peripheral edema. Within 30 days, the readmission rate was only 2.6% (1 new pacemaker implanted). Combining the unique structure of the K-Clip, we attribute the benefits to the following points. 1) The thin tissue of the TV leaflet, complicated 3D anatomical structure, the large asymmetric annulus, and existence of imaging complications,^21^ leading to difficulties and complications in repairing the leaflet. The K-Clip system was targeted for the operation of the valve ring. The structure of the TV ring is firm, and the hinge area of the tricuspid annulus is relatively broad, simple to image on ultrasound, and easy to operate, has a high in safety factor and extensive clamping range and can be adjusted repeatedly. Additionally, the same position could be clamped multiple times without causing considerable annular damage. 2) Most of the interventional TV devices need to enter the ventricle for operation, which leads to complications such as chordal entanglement, ventricular perforation, and valve leaflet damage, providing safety hazards, whereas the K-Clip only needs to be operated in the right atrium. Entering the ventricle was not required, the operation was safer and calmer, and the incidence of complications was low. 3) The K-Clip system is relatively simple to operate and has a short operation time and learning curve. 4) Corkscrew and clips are highly defined under ultrasound and digital subtraction angiography, which are ideal for communication, teaching, and training between operators and cardiologists.

The K-Clip is a type of tricuspid annuloplasty that differs from the 3 existing transcatheter annuloplasty procedures. The Cardioband system is comparable with the surgical implantation of an annuloplasty ring. The TRI-REPAIR research revealed that the overall prevalence of perioperative major serious adverse events was 13.3%; during the 30-day follow-up period, 2 people died; and other safety events included stroke, bleeding, coronary artery complications, renal failure, and implantation of a permanent pacemaker.^18^ The TriAlign device imitates the principles of Kay’s technique. The SCOUT trial demonstrated that at 30 days, the device success rate was 80%,^22^ and the detachment of one pledget was a crucial issue; however, the device is no longer accessible and developed. The TriCinch, similar to the TriAlign system, has a device success rate of 81%, bleeding, and anchor detachments are major problems, and this is no longer accessible for clinical use. Compared with the TRILUMINATE Pivotal Trial^8^ (the first prospective randomized trial of percutaneous tricuspid transcatheter edge-to-edge repair for severe TR), the population in our study was younger, the proportion of NYHA functional class III/IV was higher, and the results of the 2 studies regarding the 30-day follow-up were similar. It can be observed from the comparison that the special design of the K-Clip guarantees the safety and reliability (the dual anchoring effect of the anchor device and clip device, tackling annular dilatation directly) of the operation. Additionally, the K-Clip, an annuloplasty method, has no impact on the simultaneous/subsequent inclusion of other transcatheter TV repair methods such as TriClip, and the K-Clip has the potential to be a complementary therapy to leaflet devices, especially because of the fact that it mostly addresses the posterior part of the annulus, which is different from transcatheter edge-to-edge repair.

Currently, it is widely accepted that uncorrected severe TR enhances the risk of death and reduces the survival rate,^4^ and extremely few patients with severe TR receive effective treatment. It can be observed from this research that the K-Clip is a safe and effective treatment, excluding one case of new pacemaker implantation; almost no other adverse events occurred, and the patients showed significant clinical benefits. Thus, we recommend transcatheter treatment for moderate-to-severe TR using the K-Clip device. This will provide better advantages for high-risk patients in whom conventional treatments fail.

### Study limitations

This was not a randomized controlled study; hence, the conclusions and applicability cannot be fully encompassed in all patients with TR. For instance, a lead pacemaker causing TR was excluded from the study because the inclusion and exclusion criteria were established during patient selection. It is noted that the pacemaker lead-induced TR accounted for 10% to 25%, which is an extremely high proportion, and the left heart function of the enrolled patients is relatively good, making the outcomes of this investigation feasible and likely not applicable to all types of patients with TR. Although the effect of the clip on the RCA does not seem to be short-term issues, it is also an issue that we need to worry about. Additionally, concerning the grading technique of TR, we adopted the latest updated 6-grade scheme by Hahn et al,^13^ different from the 5-grade scheme used in previous TR studies. This offers some complications for the horizontal comparison of reflux grade decline among different studies. Finally, we anticipate the 1-year follow-up outcomes and multicenter research findings, hoping to provide new treatments to patients with TR.

## Conclusions

This study demonstrates that the K-Clip device is technically feasible, safe, and effective in patients with severe FTR. Short-term follow-up revealed that transcatheter tricuspid annuloplasty using the K-Clip system was associated with a 30-day reduction in TR and enhanced cardiac function and quality of life. In addition, further studies are required to determine the long-term outcomes.

PERSPECTIVES **COMPETENCY IN PATIENT CARE:** With the support of well-designed products, transcatheter tricuspid annuloplasty is safe and reliable. Short-term follow-up shows improvements in TR, cardiac function, and quality of life.

**TRANSLATIONAL OUTLOOK:** Long-term follow-up data and multicenter clinical research results are expected to improve the outcomes of patients with TR.

## Funding support and author disclosures

This clinical study was funded by Huihe Medical Technology, Shanghai, China. Dr Li has received funding from the 10.13039/100014717National Natural Science Foundation of China (No. 82271812). Dr Xu has received funding from the 10.13039/100014717National Natural Science Foundation of China (No. 82200269). Dr Ni has received funding from Science Technology Department of Zhejiang Province (No. 2023C03087). All other authors have reported that they have no relationships relevant to the contents of this paper to disclose.
