# Supplementary material for: 30-Day Outcomes of Transcatheter Tricuspid Annuloplasty With the K-Clip System: A Single-Center, Observational Study
Source: JACC Adv. 2023 Oct 27;2(9):100671. doi: 10.1016/j.jacadv.2023.100671 (PMC11198610; doi:10.1016/j.jacadv.2023.100671)
Supplement: Supplemental Tables 1-4 [file mmc1.docx]

| Supplemental Table 1 Exclusion criteria of TriStar With K-Clip^®^ Transcatheter Annuloplasty System |
| --- |
| Pulmonary artery systolic blood pressure ≥55mmHg |
| Tricuspid annular plane systolic excursion ≤ 14mm |
| Tricuspid stenosis, calcification of the posterior tricuspid annulus or the chordae tendineae |
| Tenting height ≥10 mm |
| Intended annular proximity to right coronary artery (≤4mm) |
| Leaflet prominent flail or prolapse |
| Severe right ventricle enlargement (basal distance of ≥50mm) |
| Anomalous jugular venous anatomy |
| Intracardiac mass |
| History of tricuspid prosthetic valve or prosthetic ring |
| Previously implanted with lead pacemaker |
| Active infection such as endocarditis or active rheumatic heart disease |
| Inability to carry out transesophageal echocardiography or transthoracic echocardiography with moderate or more substantial aortic stenosis |
| Mitral stenosis, aortic regurgitation or patients having mitral regurgitation |
| Patients having an active peptic ulcer or active gastrointestinal bleeding |
| Myocardial infarction within the past 1 month |
| Severe end-stage disease (eg, malignancy, severe lung disease, liver disease, renal failure) |
| A life expectancy of less than one year |
| Ebstein syndrome |
| Patients with known allergies or contraindications to the raw materials or drugs of the test product (such as antiplatelet drugs, anticoagulant drugs) |
| Alcohol, drug or drug addiction |

| Supplemental Table 2 Baseline Blood Test of the Population (n = 39) | |
| --- | --- |
| Blood test | |
| Hemoglobin, g/L | 124.8 ± 16.2 |
| B-type natriuretic peptide, pg/mL | 168.7 (102.3-263.3) |
| N-terminal pro–B-type natriuretic peptide, pg/mL | 798.0 (518.0-1530.0) |
| Sodium, mmol/L | 140.5 ± 3.8 |
| Creatinine, umol/L | 81.6 ± 26.5 |
| eGFR, mL/min/1.73 m^2^ | 68.3 ± 18.9 |
| Albumin, g/L | 51.1 ± 59.4 |
| Total protein, g/L | 69.3 ± 7.9 |
| AST, U/L | 26.3 ± 9.9 |
| ALT, U/L | 19.3 ± 16.1 |
| Values are median (interquartile range) or n (%) or mean ± SD. eGFR = estimated glomerular filtration rate, AST = aspartate aminotransferase, ALT = alanine aminotransferase. | |

| Supplemental Table 3 Baseline Echocardiographic Findings (n = 39) | |
| --- | --- |
| TR severity | |
| <Grade 4 | 0 (0%) |
| Grade 4 | 22 (56.4%) |
| Grade 5 | 8 (20.5%) |
| Grade 6 | 9 (23.1%) |
| RA upper to lower diameter baseline, cm | 6.7 ± 1.4 |
| RA left to right diameter, cm | 5.6 ± 1.2 |
| Systolic pulmonary artery pressure, mmHg | 42.2 ± 13.4 |
| TR coaptation gaps, mm | 6.0 ± 2.6 |
| TR vena contracta width, mm | 12.7 ± 5.7 |
| TV EROA, mm^2^ | 99.5 ± 77.8 |
| Vena contracta area, cm^2^ | 0.9 (0.7-1.6) |
| Regurgitant volume, mL | 71.1 ± 38.7 |
| Anteroposterior diameter of tricuspid valve annulus, mm | 40.9 ± 6.1 |
| Septolateral diameter of tricuspid valve annulus, mm | 41.9 ± 6.9 |
| Diastolic TV opening area, cm^2^ | 15.0 ± 5.2 |
| TV ring circumference, mm | 137.0 ± 23.2 |
| RV diameter base, mm | 45.8 ± 7.6 |
| Fractional area change, % | 43.4 ± 7.9 |
| Inferior vena cava diameter, mm | 22.0 ± 7.1 |
| Inferior vena cava variability, % | 33.9 ± 14.9 |
| Hepatic vein systolic flow reversal, m/s | 0.5 ± 0.2 |
| TAPSE, mm | 18.4 ± 3.7 |
| MR severity | |
| Mechanical valve | 17 (43.6%) |
| No MR | 0 (0%) |
| Grade 1 (mild) | 9 (23.1%) |
| Grade 2 (moderate) | 10 (25.6%) |
| Grade 3 (moderate-severe) | 3 (7.7%) |
| Left ventricular ejection fraction, % | 62.2 ± 7.8 |
| Values are median (interquartile range) or n (%) or mean ± SD. TR = tricuspid regurgitation, TV = tricuspid valve, EROA = effective regurgitant orifice area, RV = right ventricle, TAPSE = tricuspid annular plane systolic excursion, MR = mitral regurgitation. | |

| Supplemental Table 4 Baseline CTA annular measurements (end-diastolic) (n = 39) | |
| --- | --- |
| Diastolic TV opening area, mm^2^ | 1,714.8 ± 481.9 |
| TV ring circumference, mm | 149.4 ± 20.7 |
| Septolateral diameter of tricuspid valve annulus, mm | 46.8 ± 9.3 |
| Number of landing zone for K-Clip | 1.9 ± 0.7 |
| Annular-Right coronary artery distance（potential landing zones） | |
| Posteroseptal commisure, mm | 15.5 ± 5.2 |
| Midpoint of posterior valve annulus, mm | 6.4 ± 3.5 |
| Anteroposterior commissure, mm | 8.5 ± 3.2 |
| Values are mean ± SD. CTA = computed tomographic angiography, TV = tricuspid valve. | |
